# Blue‐Light Filtering Increases the Brightness of Natural Images in Pseudophakic Adults

**DOI:** 10.1155/joph/6653843

**Published:** 2026-02-03

**Authors:** Billy R. Hammond, Jacob B. Harth, Yaw Buabeng, Lisa M. Renzi-Hammond

**Affiliations:** ^1^ Behavioral and Brain Sciences Program, University of Georgia, Athens, Georgia, USA, uga.edu; ^2^ Institute of Gerontology, University of Georgia, Athens, Georgia, USA, uga.edu

## Abstract

**Purpose:**

Several studies have suggested that blue‐light filtering (BLF) can enhance the perception of brightness. Evidence for this effect, however, in pseudophakic patients, particularly using natural images, is lacking. The present study tested whether a common BLF spectral profile, often used in intraocular lens (IOL) designs, would influence brightness perception of natural images in pseudophakic individuals.

**Methods:**

Forty pseudophakic participants (*M* = 71.15 ± 2.27 years) with 20/40 or better best‐corrected visual acuity implanted with clear IOL implants completed a brightness matching task. Participants viewed a series of natural images through both a clear extraocular filter and a BLF test lens. The test lens was designed to approximate a typical BLF IOL transmission profile. Filter conditions were counterbalanced and randomized. Participants adjusted a short‐wave deficient comparison field until the natural scene and the comparison fields were perceived as equally bright. Matched luminance values (log relative energy, LRE) were recorded across six trials per image. Images were achromatic or short‐, mid‐, and long‐wave dominant.

**Results:**

Averaged across all images, the clear lens ($\overset{¯}{X}$ = 2.74 ± 0.14) resulted in significantly lower (*t*_[78]_ = −2.529, *p* = 0.007) LRE values compared to the BLF test lens ($\overset{¯}{X}$ = 2.82 ± 0.15) indicating a modest (∼17%) increase in perceived brightness with the BLF lens. This effect was observed for four of five natural images tested and was not dependent on image contrast or chromatic content.

**Conclusions:**

The BLF produced a significant and consistent increase in perceived brightness in pseudophakic adults. These findings provide direct psychophysical evidence that clinically relevant BLF profiles can influence brightness under natural viewing conditions. Information of this type is necessary for the evaluation of BLF IOL designs and their effects on functional visual experiences following cataract surgery.

## 1. Introduction

Brightness, the judgement of how the luminance of an object appears to an observer, is subjective but surprisingly lawful. Mathematically, for a given luminance *L*, the perceived brightness of an image does not increase linearly with intensity. Rather, it is best described by the power‐law relationship originally formalized by Stanley Stevens [1]: *B* ∝ *L* ^*n*^ where *B* represents perceived brightness, *L* is the luminance, and the exponent (*n*) is defined by the visual judgement (around 0.3 to 0.5 for brightness perception in typical conditions).

Although brightness scales with luminance, it is known to be moderated by multiple factors [2]. These factors [3] include chromatic contrast, adaptation state, and the visual context (e.g., surrounds). In clinical optics, an additional and less intuitive factor is spectral filtering, particularly blue‐light filtering (BLF). For example, a recent study [4] showed that a plasmonic BLF could block 25%–30% of short‐wave light 400–500 nm) without degrading key optical performance metrics (like the modulation transfer function or optical aberrations). A number of studies have also suggested that filtering short‐wave light can lead to increases in perceived brightness [5–7]. Hence, the question: how can reducing light input through filtering increase the perception of luminance?

**Figure 1:**
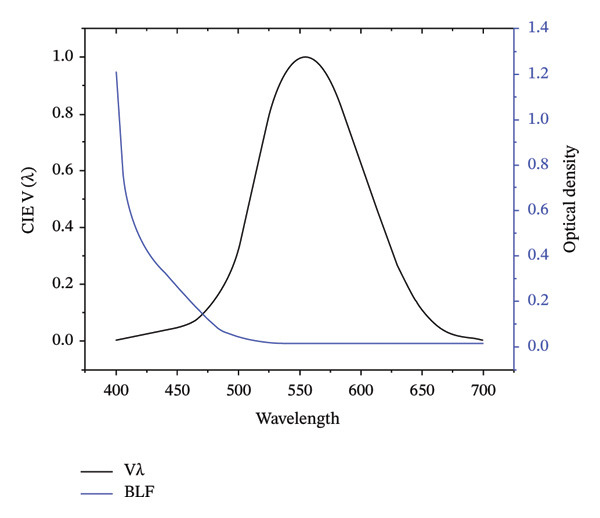
The optical density (black line) of common BLFs (Alcon Natural/Clareon) and the photopic spectral sensitivity curve, black line (CIE Photopic V(λ) [also known as CIE VM(λ)]).

BLF does not appear to actually reduce luminance in a particularly significant way, particularly during the day. This is shown in Figure 1. As seen in the figure, the short‐wave portion of the photopic curve is a relatively small part of the overall curve. Common BLF, such as those found in intraocular lens (IOL) implants (e.g., Figure 1 shows the spectra for AcySof Natural and Clareon, common BLFs used in IOL marketed as BLF) filter the 400–500 nm waveband but absorb most heavily from a narrower waveband, between 400 and 440 nm.

From a clinical perspective, IOL selection following cataract surgery increasingly considers not only optical quality and photoprotection but also patient‐reported visual experience. Understanding whether commonly used BLF IOLs systematically alter perceived brightness under natural viewing conditions is therefore relevant to both postoperative counseling and IOL design decisions.

We also know that sensitivity (i.e., V[λ]) and color perception are not altered by natural intraocular BLF such as macular pigment (MP), at least for young adults [8, 9]. Even older individuals with an optically dense MP and crystalline lens (i.e., a combined short‐wave absorbance over a log unit, less than 5% transmission at peak absorbance) do not appear to have reduced short‐wave sensitivity or altered color perception [10]. This is because the visual system effectively compensates by increasing the gain of the system to offset the filtering [8, 9]. Compensation for sensitivity loss due to intraocular filtering may be reduced in older individuals, but brightness perception seems to be maintained (despite even dense yellowing of the lens). For example, Kraft and Werner [11] found that spectral sensitivity decreased with age because of increased filtering by the lens (and likely neural loss), but brightness perception across wavelengths did not change.

Maintaining sensitivity despite light loss due to filtering is different, however, than enhancing brightness. Whether compensatory mechanisms can result in a net increase in perceived luminance remains unresolved. Can compensating for light loss due to filtering actually increase the overall luminance signal? Kelly [5] originally argued that BLF brought a small amount of rods “online,” effectively boosting the overall luminance signal by ∼40%. This increase was negligible during the cone plateau of the dark adaptation curve (when rods were inactive/bleached), suggesting that the effect was mediated by the contribution of rod signals to the chromatic pathway. Although intriguing, the effect could be limited, i.e., it may not generalize to photopic viewing or to pseudophakic observers.

Other studies that directly measure brightness do seem to be consistent with the widespread notion that yellow filters brighten the visual field. For example, Wolffsohn et al. [6] tested 20 young participants using a questionnaire (“Is vision with this filter brighter or dimmer?”) while they viewed an outdoor scene consisting of “sky, houses, road, trees, and grass” through chromatic filters. In this scenario, only the 450 nm cutoff (step‐filter) improved brightness relative to a clear control. Luque et al. [7] tested filter effects on brightness using asymmetric matching. In this method, participants were shown two stimuli side by side, one viewed through a yellow filter (a cut‐off filter, similar to Wolffsohn et al. [6]), the other viewed without a filter. Participants adjusted the brightness of one stimulus until both stimuli appeared equally bright. Luque et al. [7] found that brightness improvement was dependent upon the surround: BLF improved brightness with blue surrounds, but BLF reduced brightness with black, white, or yellow surrounds.

Despite these findings, few studies have directly examined brightness perception in pseudophakic individuals using BLF profiles representative of modern IOL designs and naturalistic visual stimuli. This gap is notable given the widespread clinical use of BLF IOLs and ongoing debate regarding their functional visual consequences. In this study, we used a brightness‐matching technique to determine whether a common filtering profile used in IOL designs (Clareon or AcrySof natural; see Hammond et al. [12]) would influence brightness perception in pseudophakic adults. Participants viewed static natural images under controlled photopic conditions, presented side by side with an adjustable short‐wave deficient comparison scene. Images were viewed through either a clear control lens or a BLF test lens, with conditions counterbalanced and randomized. If the natural image was perceived as brighter, greater luminance would be required in the comparison field to achieve a perceptual match.

## 2. Methods

### 2.1. Participant Selection and Ethics

Our a priori power analysis suggested that a final analyzable sample of *N* = 40 participants would be needed to detect hypothesized differences between the test lenses. To achieve the final analyzable sample, 134 potential participants were recruited from two local ophthalmology practices. A total of 9 of the 134 participants initially failed the screening. Of the remaining 125 potentially eligible participants, 75 participants were unable to participate despite meeting inclusion criteria (due to issues with transportation, general lack of interest in participating in research, illness, etc.), and 10 participants who made study appointments failed to attend the study visit and were lost to follow‐up. The final analyzable sample (*N* = 40; *M* = 71.15 ± 5.82 years; 82.5% White/Caucasian; 57.5% female) had 20/40 or better binocular best‐corrected visual acuity (BCVA), were fluent in English, had no ocular pathology that would limit participation. These demographic data are shown in Table 1.

**Table 1:** Demographic and visual function characteristics of the study sample.

Variable		Percentages (%)	*n*
Gender	Man	42.5	17
	Woman	57.5	23

Race	White	82.5	33
	Black	15	6
	Asian	2.5	1

Ethnicity	Non‐Hispanic	97.4	38
	Hispanic	2.6	1

Iris lightness	Light	55.0	22
	Medium	30.0	12
	Dark	15.0	6

Iris hue	Gray	20.0	8
	Blue	32.5	13
	Green	7.5	3
	Hazel	17.5	7
	Brown	22.5	9

Best uncorrected visual acuity (OU)	20/15	5.0	2
	20/20	70.0	28
	20/30	20.0	8
	20/40	5.0	2

The study protocol was approved by Sterling IRB (Atlanta, GA; Project ID number 11865), with local context provided by the University of Georgia Human Research Protection Program. All participants provided written and verbal informed consent prior to participation.

#### 2.1.1. Study Design

A randomized, controlled, crossover design was used, where pseudophakic participants with clear IOL implants were tested under two conditions presented in random order. In one condition, brightness matches were obtained while participants viewed the natural scene and adjustable short‐wave deficient comparison field through a clear control lens. In the second condition, participants viewed these stimuli through a BLF test lens. Both lenses were mounted in adjustable spectacles, which were placed on the participants’ faces by the investigator. Our alternative hypothesis was that seeing the images through a BLF filter would result in an increase in perceived brightness when compared to a clear control lens (*A priori* H_1_: Control log‐relative energy [LRE] < Test LRE).

### 2.2. Study Lenses

Optical density for the BLF test lens filter across the visible spectrum is presented in Figure 1 below (the optical density of the clear control lens is about 0.013 over the visible range). Transmission spectra for the BLF test lens and the clear control can be found in Hammond et al. [12].

#### 2.2.1. Apparatus and Procedure

**Figure 2:**
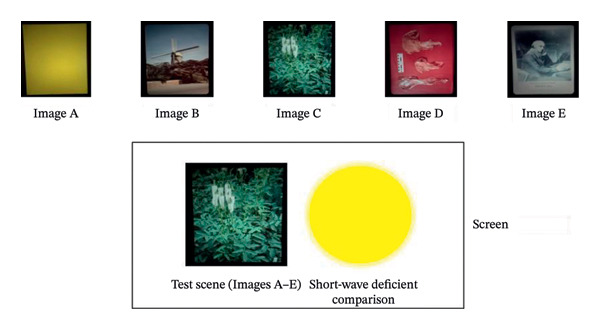
The five images that were used. These images were kept at a constant energy while the comparison field was adjusted to match in brightness.

Brightness perception was assessed using the standard heterochromatic brightness matching paradigm. Unlike the classic small bipartite field, participants compared the brightness of a short‐wave deficient yellow comparison field against a variety of natural scene images shown in Figure 2 (similar to the paradigm of Bartleson and Breneman [13]). A schematic of the apparatus used to measure brightness perception can be found in Hammond et al. (2025) [14] Natural scenes were created using rectangular photographic slides projected onto a matte white screen using a 550‐W xenon slide projector (Navitar Xenon 560). Natural scenes had approximately the same luminance (2–12 cd/m^2^ at the screen depending on the area of the scene measured) and were the same size, subtending approximately 15°deg of visual angle. The 15°deg circular comparison field was generated by a single‐channel parallel optical system consisting of a 1000‐W xenon arc lamp (ThermoOriel Instruments Lamp, model SP66921‐3016; Newport Oriel Instruments power source, model 69,920; Stratford, CT, USA), a series of achromatic lenses (the final one focusing the light onto the screen), and a yellow Corning 51,300 glass filter positioned behind an adjustable neutral density wedge. The yellow Corning filter ultimately created a field that was broadband but short‐wavelength deficient and not filtered by the BLF nor the control lens.

At the start of each test session, natural scene luminance was confirmed using an Industrial Fiber Optics Digital Photometer (model number: IF PM). Participants were seated 78 inches away from the screen, fitted with spectacle lenses that contained the clear control lens or the BLF, and the comparison field was adjusted to be either noticeably brighter or dimmer than the natural scene. Participants were asked to look through the spectacle lenses at the screen. The investigator, using the ascending and descending method of limits, then adjusted the intensity of the comparison field relative to the natural scene, until participants indicated that the comparison field and natural scene were equally bright. The LRE of the comparison field at the match was recorded. LRE is based on radiometric calibration of the test stimuli. The values are measured in energy units (e.g., watts) and then logged (base 10). A test scene that is judged as brighter by the subject would translate to a higher LRE for the matched comparison field. A minimum of three trials were completed per image, per test lens (BLF or control lens). Test lens order was randomized for each participant.

### 2.3. Statistical Analysis

Prior to hypothesis testing, Levene’s test was used to determine whether the homogeneity of variance assumption was met, and the Shapiro‐Wilk test was conducted to determine whether distributions were normal. For all scenes tested, data were normally distributed, and homogeneity of variance was maintained. Consequently, *t*‐tests were used to determine whether the BLF test lens significantly differed from the clear control lens. Given the fact that our a priori hypotheses were directional in nature (LRE for the clear control lens < LRE for the BLF lens, across all scenes), one‐tailed tests were used, with *p* < 0.05 as the criterion for significance, and *p* < 0.10 as the criterion for a statistical trend.

## 3. Results

To assess the overall effect of filtering across image types, LRE values were analyzed using a repeated‐measures analysis of variance with Filter (clear vs. BLF) and Image (A–E) as within‐subject factors. This analysis revealed a significant main effect of Filter (*F* = XXX), indicating higher perceived brightness under BLF conditions. The Filter × Image interaction was not significant, suggesting that the BLF effect was consistent across image content.

**Figure 3:**
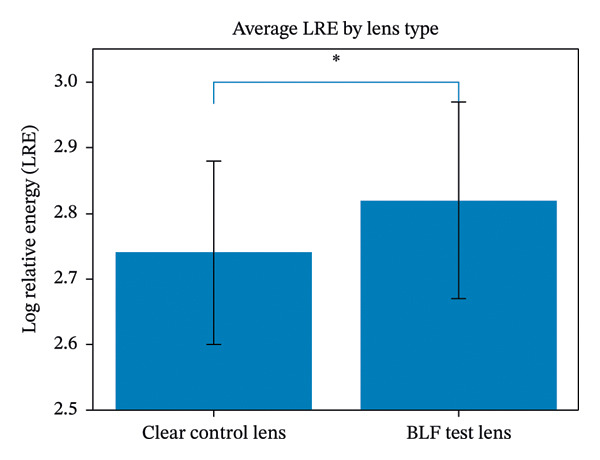
The average energy needed in the comparison field to match the brightness of the test image. The BLF lens required about 17% more energy indicating that the natural image appeared brighter.

The results based on individual images are provided in Table 2. As shown in the table, for the yellow reference (Image A), the LRE for the clear control lens ($\overset{¯}{X}$ = 2.82 ± 0.12) was significantly lower (*t*_[78]_ = −2.127, *p* = 0.018) than the LRE for the BLF test lens ($\overset{¯}{X}$ = 2.88 ± 0.12). For the blue windmill scene (Image B), there was a trend (*t*_[78]_ = −1.298, *p* < 0.10) for differences between the clear control lens ($\overset{¯}{X}$ = 2.77 ± 0.17) and the BLF test lens ($\overset{¯}{X}$ = 2.82 ± 0.19). For the image of green bushes (Image C), the LRE for the clear control lens ($\overset{¯}{X}$ = 2.71 ± 0.18) was significantly lower (*t*_[78]_ = −2.265, *p* = 0.013) than the LRE for the BLF test lens ($\overset{¯}{X}$ = 2.80 ± 0.19). For the red‐dominant scene (Image D), the LRE for the clear control lens ($\overset{¯}{X}$ = 2.68 ± 0.17) was significantly lower (*t*_[78]_ = −2.265, *p* = 0.013) than the LRE for the BLF test lens ($\overset{¯}{X}$ = 2.77 ± 0.18). For the daguerreian scene (Image E), the clear control lens LRE ($\overset{¯}{X}$ = 2.74 ± 0.16) was significantly lower (*t*_[78]_ = −3.080, *p* < 0.001) than the LRE for the BLF test lens ($\overset{¯}{X}$ = 2.85 ± 0.16). Finally, when LRE scores were averaged for the test filter across the images shown in Figure 2, the average LRE for the clear control lens ($\overset{¯}{X}$ = 2.74 ± 0.14) was significantly lower (*t*_[78]_= −2.529, *p* = 0.007) than the LRE for the BLF test lens ($\overset{¯}{X}$ = 2.82 ± 0.15). In other words, natural scenes appeared to be about 17% brighter (on a linear energy scale) when viewed through the BLF test lens than when viewed through the clear control lens. This is shown in Figure 3.

**Table 2:** Comparison of luminous relative efficiency (LRE) between clear control lens and BLF test lens across images.

Image (scene type)	Clear control M ± SD	BLF test M ± SD	*t* (df = 78)	*p*‐value	Interpretation
Image A (Yellow reference)	2.82 ± 0.12	2.88 ± 0.12	−2.127	0.018	Significant difference (BLF > Control)
Image B (Windmill)	2.77 ± 0.17	2.82 ± 0.19	−1.298	< 0.10	Trend toward difference
Image C (Green bushes)	2.71 ± 0.18	2.80 ± 0.19	−2.265	0.013	Significant difference (BLF > Control)
Image D (Red‐dominant)	2.68 ± 0.17	2.77 ± 0.18	−2.265	0.013	Significant difference (BLF > Control)
Image E (Daguerreian scene)	2.74 ± 0.16	2.85 ± 0.16	−3.080	< 0.001	Significant difference (BLF > Control)
Average (All Images)	2.74 ± 0.14	2.82 ± 0.15	−2.529	0.007	Significant difference (BLF > Control)

*Note:* Values are means (M) ± standard deviations (SD) for LRE scores. *T*‐tests were conducted for each image and for the average LRE. All comparisons were one‐tailed, with *p* < 0.05 as the criterion for significance. Across images, the BLF test lens consistently yielded higher LRE scores than the clear control lens.

## 4. Discussion

In this study the BLF test lens resulted in a significant average increase in brightness perception (about 17%) across four of the five images that were tested (see Table 2 and Figure 3). This effect seemed largely unrelated to the contrast within the figures or the degree to which the test lens filtered the image. These results are consistent with Kelly’s [5] original hypothesis that BLF might subtly recruit rod photoreceptors even under photopic conditions, boosting the total luminance signal. Typically in photopic conditions, rods are largely saturated and inactive, but a reduction in short‐wave light might reduce this saturation, allowing a modest rod contribution to augment the cone‐mediated brightness signal. An increase in matched brightness levels (LRE) with the BLF lens, across a diverse set of natural scenes, is consistent with a mechanism not tied strictly to image contrast, scene chromaticity, or the absolute reduction in short‐wave input, but rather to an adaptive recalibration of the luminance pathway. This recalibration appears to be possible even in older pseudophakic participants, as originally noted over a longer time scale by Delahunt et al. [10].

Another possible driver is pupil size. Chung and Pease [15] originally showed that wearing yellow filters resulted in a statistically significant increase in pupil size under various lighting conditions, with a more noticeable effect in dim light. The authors speculated that brightness enhancement by yellow filters could be due to the filters reducing the overall luminance reaching the eye prompting the pupils to dilate to maintain adequate retinal illumination. The authors used a Corning Photochromic Filter (CPF) 550 which is a step filter that blocks most of the light shorter than 550 nm which is quite different than the spectral filtering we used in our study. The effect of a BLF on pupil size is primarily driven by absorbance centered at 480 nm [16], and the BLF we used had minimal absorbance at that wavelength (see Figure 1). Nonetheless, a limitation of this study is that *w* did not measure pupil size, and therefore contributions to the brightness effect from pupil change cannot be ruled out.

## 5. Conclusion

In summary, this study demonstrates that BLF lenses can significantly enhance brightness perception. This effect appears largely independent of image contrast, chromaticity, or the degree of spectral filtering, suggesting a more complex underlying mechanism. The findings align with Kelly’s hypothesis that BLF may subtly reduce rod saturation under photopic conditions, allowing rods to modestly augment the cone‐mediated luminance signal.

This adaptive recalibration of the luminance pathway, observed even in older pseudophakic participants, suggests that selective spectral filtering can influence functional visual experience beyond what is predicted by conventional photometric measures. Such effects may be particularly relevant for individuals with aging visual systems or IOL implants, in whom luminance processing and neural compensation may already be altered.

Future research should examine brightness perception under dynamic viewing conditions and directly assess the contributions of pupil dynamics, rod‐cone interactions, and melanopsin‐mediated pathways. Longitudinal studies comparing implanted BLF and non‐BLF IOLs, as well as investigations incorporating objective physiological measures, may help clarify the mechanisms underlying brightness enhancement and inform the design of next‐generation adaptive optical devices.

## Author Contributions

Billy R. Hammond and Lisa M. Renzi‐Hammond were involved in the conception and design of the study, data acquisition, data analysis and interpretation, and manuscript preparation. Yaw Buabeng participated in data acquisition and manuscript editing. Jacob B. Harth participated in data acquisition, design, supervision, and manuscript editing.

## Funding

This project was supported by Alcon Laboratories.

## Disclosure

All authors read and approved the final manuscript.

## Conflicts of Interest

The authors declare no conflicts of interest.

## Data Availability

The data that support the findings of this study are available from the corresponding author upon reasonable request.
